# A novel patient-derived organoids-based xenografts model for preclinical drug response testing in patients with colorectal liver metastases

**DOI:** 10.1186/s12967-020-02407-8

**Published:** 2020-06-12

**Authors:** Mi Jian, Li Ren, Guodong He, Qi Lin, Wentao Tang, Yijiao Chen, Jingwen Chen, Tianyu Liu, Meiling Ji, Ye Wei, Wenju Chang, Jianmin Xu

**Affiliations:** 1 Department of General Surgery, Zhongshan Hospital, Fudan University, No. 180 Fenglin Road, Shanghai, 200030 China; 2 Shanghai Engineering Research Center of Colorectal Cancer Minimally Invasive, Shanghai, 200030 China

**Keywords:** Colorectal cancer, Liver metastases, Animal model, Organoids

## Abstract

**Backgrounds:**

Cancer-related mortality in patients with colorectal cancer (CRC) is predominantly caused by development of colorectal liver metastases (CLMs). How to screen the sensitive chemotherapy and targeted therapy is the key element to improve the prognosis of CLMs patients. The study aims to develop patient-derived organoids-based xenografted liver metastases (PDOX-LM) model of CRC, to recapitulate the clinical drug response.

**Methods:**

We transplanted human CRC primary tumor derived organoids in murine spleen to obtain xenografted liver metastases in murine liver. Immunohistochemistry (IHC) staining, whole-exome and RNA sequencing, and drug response testing were utilized to identify the homogeneity in biological and genetic characteristics, and drug response between the PDOX-LM models and donor liver metastases.

**Results:**

We successfully established PDOX-LM models from patients with CLMs. IHC staining showed that positive expression of CEA, Ki67, VEGF, FGFR2 in donor liver metastases were also well preserved in matched xenografted liver metastases. Whole-exon sequencing and transcriptome analysis showed that both xenografted and donor liver metastases were highly concordant in somatic variants (≥ 0.90 frequency of concordance) and co-expression of driver genes (Pearson’s correlation coefficient reach up to 0.99, *P* = 0.001). Furthermore, drug response testing showed that the PDOX-LM models can closely recapitulated the clinical response to mFOLFOX6 regiments.

**Conclusions:**

This PDOX-LM model provides a more convenient and informative platform for preclinical testing of individual tumors by retaining the histologic and genetic features of donor liver metastases. This technology holds great promise to predict treatment sensitivity for patients with CLMs undergoing chemotherapy.

## Background

Colorectal cancer (CRC) represents the third leading cause of cancer-related deaths worldwide [1]. Cancer-related mortality is predominantly caused by development of colorectal liver metastases (CLMs), which occurs in approximately 50% of all patients during the course of their illness [2]. Despite application of target agents and advanced therapeutic schedule, the median survival of patients with metastatic CRC (mCRC) is limited at approximately 30 months [3]. Therefore, how to screen the sensitive drugs for CLMs is critical to improve survival of these patients.

The pre-clinical animal models play critical roles in precision medicine, by elucidating biomarkers that predict drug response and identify patients who are most likely to benefit from a specific treatment [4, 5]. An ideal animal model should recapitulate the molecular, histopathological, and etiological characteristics of the donor tumors [6]. Currently, a number of animal models have evolved toward sophisticated systems that mimic the pathology of CRC. In particular, patient-derived xenografts (PDX) models and genetically engineered mouse models (GEMMs) have been widely used in metastases research [4, 7]. On one hand, PDX models have advantages in replicating the characteristics and genetic diversity of the donor tumor, and these models provide platform for pre-clinical drug response testing. However, the metastatic PDX models are time-consuming, expensive, and technically challenging [8]. On the other hand, the GEMMs are popular in the research of carcinogenic progress and the mechanisms of specific cancer-related genes. However, they usually cannot fully reproduce the genetic complexity of human tumors in the clinical practice.

Recently, the emerging organoid culture technology allows for cancer cells to be grown in a 3-dimensional matrix and have been a critical progress in cancer research [9]. Prior studies have demonstrated that patient-derived cancer organoids can be easily maintained and manipulated in vitro and faithfully recapitulate characteristics of the patient tissues [9, 10]. The establishment of living tumor organoid biobanks offers a platform for high-throughput drug screens [9]. Although the patient-derived organoids (PDOs) represents a powerful resource for finding effective therapeutic strategies direct to specific tumor subtypes, it does not account for the interplay between tumor cells and the surrounding tissue microenvironment, because this interplay could not be recapitulated in a dish [9–11]. Many studies have pointed to the importance of tumor microenvironment in influencing tumor cell identity and behavior [12–15], emphasizing the necessity of validating the results obtained in vitro in animal model systems.

To overcome these limitations, we have recently developed a method to transplant human CRC organoids in murine spleen to obtain xenografted liver metastases model, named patient-derived organoids-based xenografted liver metastases (PDOX-LM) model. This transplantation approach allows primary tumor formation in murine spleen and the spontaneous development of metastases in murine liver. And we further verified the homogeneity between the xenografted and donor liver metastases in histological feature, tumor-specific protein, genetic and transcriptome characterization and drug response.

## Methods

### Human tissues

Colonic tissues were obtained from the Zhongshan Hospital with informed consent and the study was approved by the ethical committee. All patients were diagnosed with colorectal cancer. From the resected colorectal segment, tumor tissue was isolated. The isolation of tumor epithelium was performed essentially as described by Hans Clevers [16].

### Organoid preparation and transplantation

Human tissue-derived organoids were obtained and cultured according to Hans Clevers [16]. The expanded organoids were harvested with Cell Recovery Solution (BD Biosciences) and suspended in Matrigel at a concentration of 1 × 10^5^ cells/ml. A total 200 μl (2 × 10^4^ cells) of Matrigel-organoid suspension was injected into the spleen of each Balb/c-nu mice. The mice were euthanized 7 weeks after xenotransplantation. The spleens, the livers, the lungs and the other abdominal organs were isolated and examined. The size of the engrafted or metastasized tumors were determined on measurement by vernier caliper. Each CRC organoid line was transplanted into four independent Balb/c-nu mice. Some samples were then stored in liquid nitrogen, and the rest of samples were formalin-fixed and embedded in paraffin for subsequent IHC analysis. All animal procedures were approved by the Animal Ethics Committee of Zhongshan hospital and complied with the Guide for the Care and Use of Laboratory Animals.

### Whole-exome sequencing and single nucleotide polymorphisms

For each sample, 250 ng DNA was sheared and subject to whole-exome sequencing using the Agilent v2 capture probe set and sequenced by HiSeq 2500 using 76 base pair reads, as previously described [17, 18]. A median 12.4 Gb of unique sequence was generated for each sample. Sequence data were locally realigned to improve sensitivity and reduce alignment artifacts prior to identification of mutations, insertions, and deletions as previously described [19–21].

### Somatic copy-number analysis

Somatic copy-number analysis was performed using segmented copy-number profiles generated from whole-exome sequencing using the SegSeq algorithm [22]. Briefly, read depth in tumor and normal pairs was calculated to provide relative copy-number ratios at each exon followed by circular binary segmentation [23]. The GISTIC2.0 algorithm [24] was used to investigate focal and arm-level copy-number changes (Additional file 2: Table S1A–E).

### RNA sequence data processing

RNA was extracted from xenografts and tumor tissues using QIAGEN RNA mini kit. RNA sample was hybridized on Affymetrix Human Gene 2.0 ST arrays. The raw CEL files were processed with Affymetrix Power Tools using the Hg19 genome build and NetAffx annotation dating from 09-30-2018. Between-array normalization was performed using rma-sketch, within APT. This resulted in an intensity matrix of 21,681 genes by 2 samples. For analysis of individual genes, data were analyzed using the R2 web application, which is freely available at http://r2.amc.nl.

### Drug response testing

PDOX-LM models based on Patient #3, #4, #5 and #6 were randomly distributed into two groups: the control group (n = 16) for intraperitoneal injection with phosphate buffer saline [PBS, 10 mM, 0.1 ml per mouse] 2 times every weeks, and the chemotherapy group (n = 16) for intraperitoneal injection with the combination of oxaliplatin (0.4 mg/ml PBS) and 5-Fu (4 mg/ml PBS) [0.1 ml per mouse] 2 times every week. All intraperitoneal injection started from the 7th weeks since the splenic xenotransplantation of mCRC PDOs, and last for 4 weeks. In addition, the mice in chemotherapy group were further divided into PR group and PD group according to the clinical drug response of the donor patients. Supplementary methods were detail showed in the Additional file 3, and the primary antibodies used for IF or IHC staining were showed in the Table S2.

## Results

### Protocol of establishment and analysis of a PDOX-LM model

Here we presented a detailed description of the procedure for the PDOX-LM model (Additional file 1: Figure S1). This approach allowed the formation of xenografted metastases in murine liver, but not in extra-hepatic sites. By immunohistochemical (IHC) staining, whole-exome and RNA sequence, and drug response testing, we verified that the xenografted liver metastases formed by organoids of primary tumor recapitulated similar mutations, gene expression and drug response of the donor liver metastases.

### Establishment of patient-derived organoids from primary tumors of CRC patients

We adopted the Wnt-dependency of cancer stem cell to selectively expand tumor organoids, according to the previous reports [10, 16, 25]. In this study, surgically resected tissues were obtained from previously untreated CRC patients. Primary tumor derived organoids were all successfully generated from the samples of 6 metastatic mCRC and 6 localized CRC cases (Table 1). To develop a long-term expandable organoid culture, the combination of epithelial growth factor (EGF) and the Wnt amplifier R-spondin1 was essential to add in the culture medium [8, 14, 15].

**Table 1:** Clinicopathology of patients used for patient-derived organoids

Patient no.	Age, yr	Gender	CEA, ng/ml	Tumor location	TNM stage	Metastatic organ
mCRC						
1#	60	Female	2644.3	Sigmoid	IV	Liver
2#	61	Male	122.4	Rectum	IV	Liver, Lung
3#	64	Male	546.0	Ascending	IV	Liver
4#	69	Male	3555.4	Rectum	IV	Liver
5#	64	Male	46.2	Descending	IV	Liver
6#	64	Female	3.2	Ascending	IV	Liver, Lung
CRC						
7#	69	Male	77.3	Rectum	III	None
8#	64	Male	14.6	Descending	III	None
9#	67	Male	262.8	Sigmoid	III	None
10#	64	Female	5.3	Ascending	III	None
11#	65	Female	36.6	Rectum	II	None
12#	69	Male	4.4	Sigmoid	II	None

**Fig. 1:**
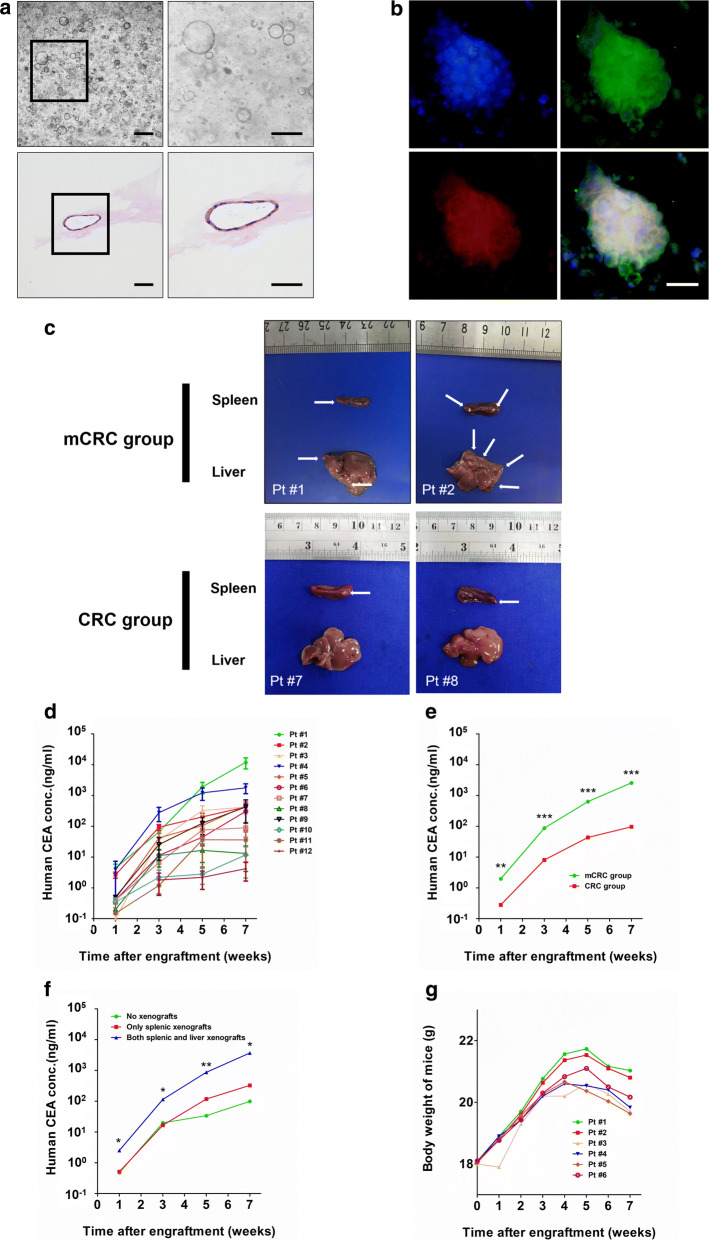
Establishment of the PDOX-LM model. **a** Preparation of primary tumor derived organoids for splenic injection. Representative images of primary tumor-derived organoids under the light microscope (above) and the morphologic feature of organoids were observed by hematoxylin and eosin (H&E) staining (below), Bar = 200 μm. **b** Representative live confocal images of a Lgr5, E-cadherin dual positive organoids cultures in Matrigel (BD science). Blue represents nuclear, which is staining by Hoechst 33342 (left, above). Green represents cell membrane of lgr5-Positive organoids, which indicated existence of the stem cell property (right, above). Red represents cell membrane of the E-cadherin-positive organoids, which indicated the cell in organoids were rooted from epithelial source (left, below). Representative image of Lgr5 and E-cadherin dual positive organoids were also showed (right, below). Bar = 150 μm. **c** Xenografted liver metastases by a splenic injection of primary tumor derived organoids. Representative pictures of splenic xenografts and xenografted liver metastases of patient #1(left, above) and #2(right above) in the mice models. White tumor xenografts were labeled(arrowhead). In picture of patient #1, only 1 xenografts in murine spleen and 2 metastatic xenografts in the murine liver were detected under macrography. In picture of patient #2, 2 xenografts in murine spleen and 4 metastatic xenografts in the murine liver were detected under macrography. Based on organoids derived from localized CRC, the xenografts were only observed in the spleen but not in the liver in mice models. Representative picture of splenic xenografts of patient #7 (left, below) and #8 (right, below) were showed. **d** Human CEA in murine blood. CEA,a serum biomarker expressed from CRC cells. Quantitative ELISA with human specific antibodies. **e** Human CEA were compared between the mCRC group (n = 24) and CRC group (n = 24). NOTE: *p value ≤ 0.05 and **p-value ≤ 0.01 when compared with CRC group; **f** Human CEA were also compared among the ‘No xenografts’ group (n = 8), the ‘Only splenic xenografts’ group (n = 25) and the ‘Both splenic and liver xenografts’ group (n = 15). NOTE: *p-value ≤ 0.05, **p-value ≤ 0.01, and ***p-value ≤ 0.001, respectively, when compared with ‘No xenografts’ group; **g** Body weight of mice after splenic xenotransplantation. After a mean period of 6 weeks after splenic xenotransplantation, a decrease in body weight of 21 mice (21/24, 87.5%) were observed

We showed the number of CRC organoids varied among different patient samples, with some tumors rendering thousands of organoids whereas others yielding only 10–20 primary organoids, which was similar with the previous studies [16]. Notably, the tumor organoids derived from mCRC cases demonstrated a higher proliferating ability (at least 200 organoids or 1 × 10^5^ cells/ml Matrigel) than those derived from localized CRC cases (a median of 40 organoids or 5 × 10^4^ cells/ml Matrigel) (Fig. 1a).

And we also identified the histologic features of CRC PDOs by hematoxylin and eosin (H&E) staining and characteristic biomarkers by immunofluorescence (IF) staining (Fig. 1b). In all cases, the cultured organoids were similar to the matched donor tumors in secondary architecture, nuclear pleomorphism, nuclear to cytoplasmic ratio, presence of prominent nucleoli, and mitotic rate. The CRC organoids commonly developed crypt-like structures reminiscent of the malignant glands seen within the patient’s cancer (Fig. 1a). In addition, these key phenotypic characteristics, such as lgr5 and E-cadherin, are maintained across passages. According to previous researches, Lgr5, an intestinal stem cell-specific membrane protein, confirmed the stemness of organoids [16], and positive expression of E-cadherin confirmed the epithelial source of organoids (Fig. 1b).

### Establishment of PDOX-LM models

After cutting a freshly procured primary CRC tumor specimen into small tissue blocks (~ 8 mm^3^), we obtained millions of single cells and some cell clusters by physical and chemical separation. Then we cultured the tumor organoids in vitro according to the protocols. After expanding, the organoids (1 × 10^5^ cells/ml Matrigel) of low-passage (≤ 6 passage) were engrafted into the murine spleens. In brief, we cut through the abdominal walls along the costal margin, and immediately injected 200 μl Matrigel-organoids suspension into the bottom of murine spleen. Following two factors may account for the successful establishment of PDOX-LM models: successful expanding organoids in vitro and immediate stanch of copious bleeding from murine spleen.

The CRC organoids derived from the primary tumors of 6 mCRC and 6 localized CRC cases were transplanted into the spleen of balb/c-nu male mice. Interestingly, we observed that the organoids of mCRC source other than localized CRC source successfully formed macrometastatic colonies (≥ 1 mm in size) in the livers [26]. In addition, using this transplanting method, macrometastatic lesion could only be found in murine livers, but not in extra-hepatic sites (Fig. 1c).

In terms of the dynamic monitoring marker in CRC, serum Carcinoembryonic antigen (CEA) is often used as a typical tumor marker [27]. The in vivo study showed that, after transplantation, we monitored human CEA levels in murine blood dynamically. Human CEA were detectable in murine blood 1 week after transplantation, with concentrations progressively increasing over the following weeks (Fig. 1d). We observed that the serum CEA in PDOX-LM models of mCRC source was higher than that in models of localized CRC source (Fig. 1e). And the serum CEA levels in successfully established PDOX-LM models were higher than that of the mouse models without xenografted liver metastases (Fig. 1f). The serum CEA levels were comparable to those detected in human cancer patients, reaching up to 10^4^ ng/ml (Table 2).

**Table 2:** Pathology of PDO-based xenografted liver metastases (PDOX-LM) models

Patient no.	Mean CEA, ng/ml			No. of grafts in spleen/total no. of grafts (%)	No. of grafts in murine liver/total no. of grafts (%)	No. of xenografted liver metastases, median (range)
	3-week	5-week	7-week			
mCRC						
1#	66.4	1834.8	12066.8	4/4 (100)	3/4 (75)	2 (2, 2)
2#	93.2	202.4	424.6	3/4 (75)	3/4 (75)	2 (2, 4)
3#	36.0	326.0	422.0	3/4 (75)	3/4 (75)	2 (2, 3)
4#	277.8	1226.4	1775.4	4/4 (100)	3/4 (75)	3 (2, 4)
5#	42.2	106.0	322.2	4/4 (100)	2/4 (50)	3.5 (2, 5)
6#	11.4	44.2	306.6	4/4 (100)	1/4 (25)	4 (4, 4)
CRC						
7#	6.4	76.0	89.4	4/4 (100)	0/4 (0)	/
8#	11.2	16.6	13.2	3/4 (75)	0/4 (0)	/
9#	25.6	126.8	424.4	3/4 (75)	0/4 (0)	/
10#	2.2	2.8	11.8	2/4 (50)	0/4 (0)	/
11#	1.2	36.6	36.2	4/4 (100)	0/4 (0)	/
12#	1.8	2.2	4.2	2/4 (50)	0/4 (0)	/

In addition, to explore the index to monitor the establishment of PDOX-LM models, we monitored the body weight of mice after transplantation dynamically. We observed that 8 mice (8/24, 33.3%) had a weight loss at 5th week, and 21 mice (21/24, 87.5%) had a weight loss at 6th week (Fig. 1g). All mice with weight loss were found to bear xenografted liver metastases when sacrificed at 7th week.

All mice were sacrificed in 7th week since transplantation in murine spleens. For transplanted organoids from Patient #1, at most two macrometastatic colonies (≥ 1 mm in size) were formed in the livers, whereas transplanted organoids from Patient #2 formed at least 4 colonies. And for transplanted organoids from Patient #3, #4, #5 and #6, the numbers of macrometastatic colonies was 2–5 in the livers (Table 2). The metastatic capacity of transplanting organoids seemed like substantially diverse.

### Histological feature and tumor-specific protein of xenografted and donor liver metastases

**Fig. 2:**
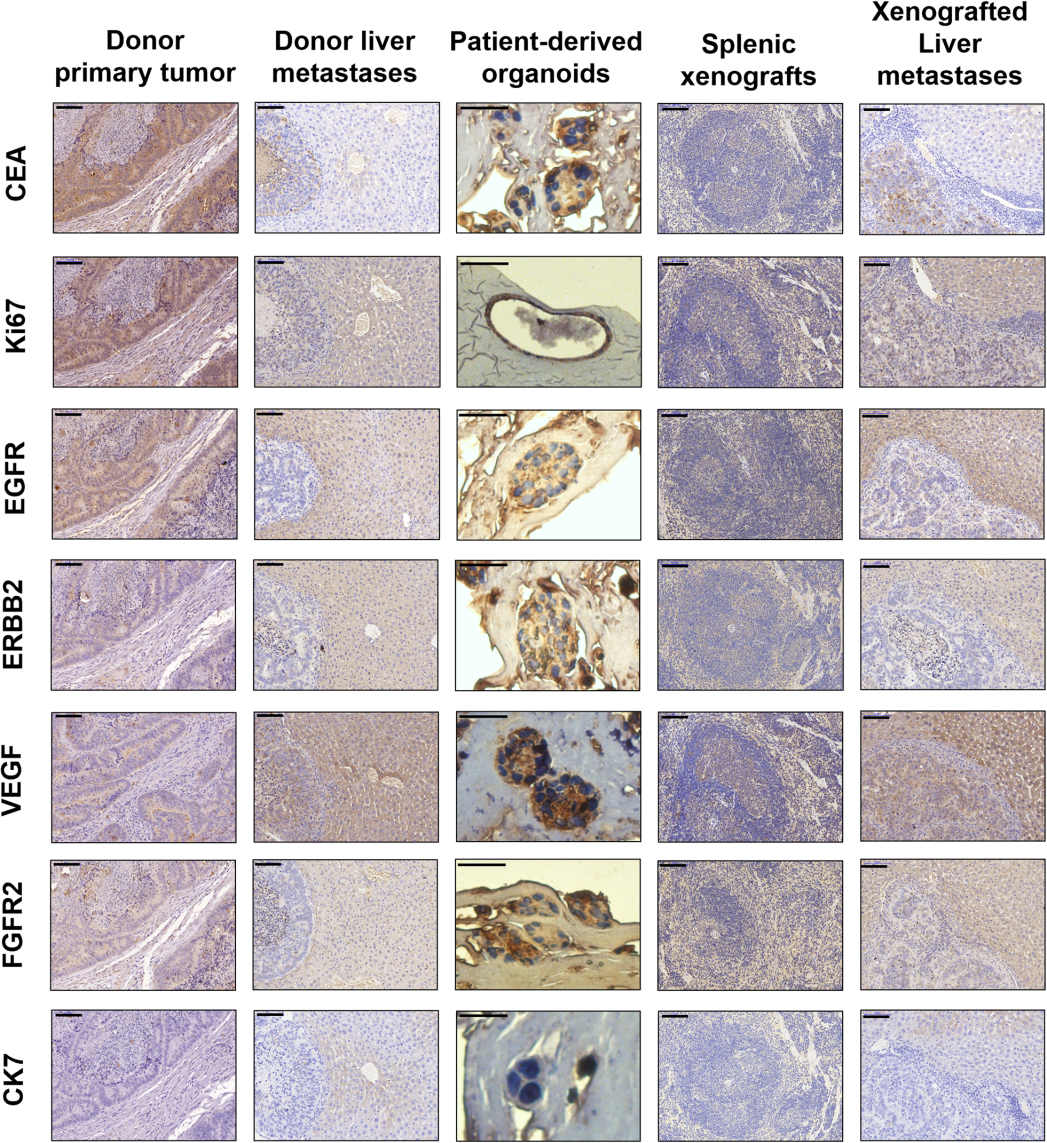
Development of a splenic transplantation of primary tumor-derived organoids to mimic liver metastases Representative CEA, Ki67, EGFR, ERBB2, VEGF, VEGFR2 and CK7 staining of a donor primary tumor, donor liver metastases, organoids, splenic xenografts, and xenografted liver metastases in patient #1 case

Histological features of the parental tumors were well preserved in these macrometastatic lesions, especially in secondary architecture, nuclear pleomorphism, nuclear to cytoplasmic ratio, presence of prominent nucleoli, and mitotic rate (Fig. 2). By means of IHC staining, positive staining of CEA, Ki67, VEGF, FGFR2 and negative staining of EGFR, CK7 of the parental lesions were also well preserved in matched organoids and xenografted liver metastases (Fig. 2). Notably, the xenografted and donor liver metastases showed similar patterns of expression of these CRC tumor-specific markers.

### Genomic characterization of xenografted and donor liver metastases

To demonstrate the maintenance of molecular features between the xenografted and donor liver metastases, we performed DNA copy-number analyses and whole-exome sequencing platforms between the xenografted and donor liver metastases [10, 16]. Matched xenografted and donor liver metastases were sequenced for 2 mCRC patients accepting simultaneous resection of primary tumors and liver metastases. And the sequencing depth were greater than 500 × median coverage across the interrogated genes.

**Fig. 3:**
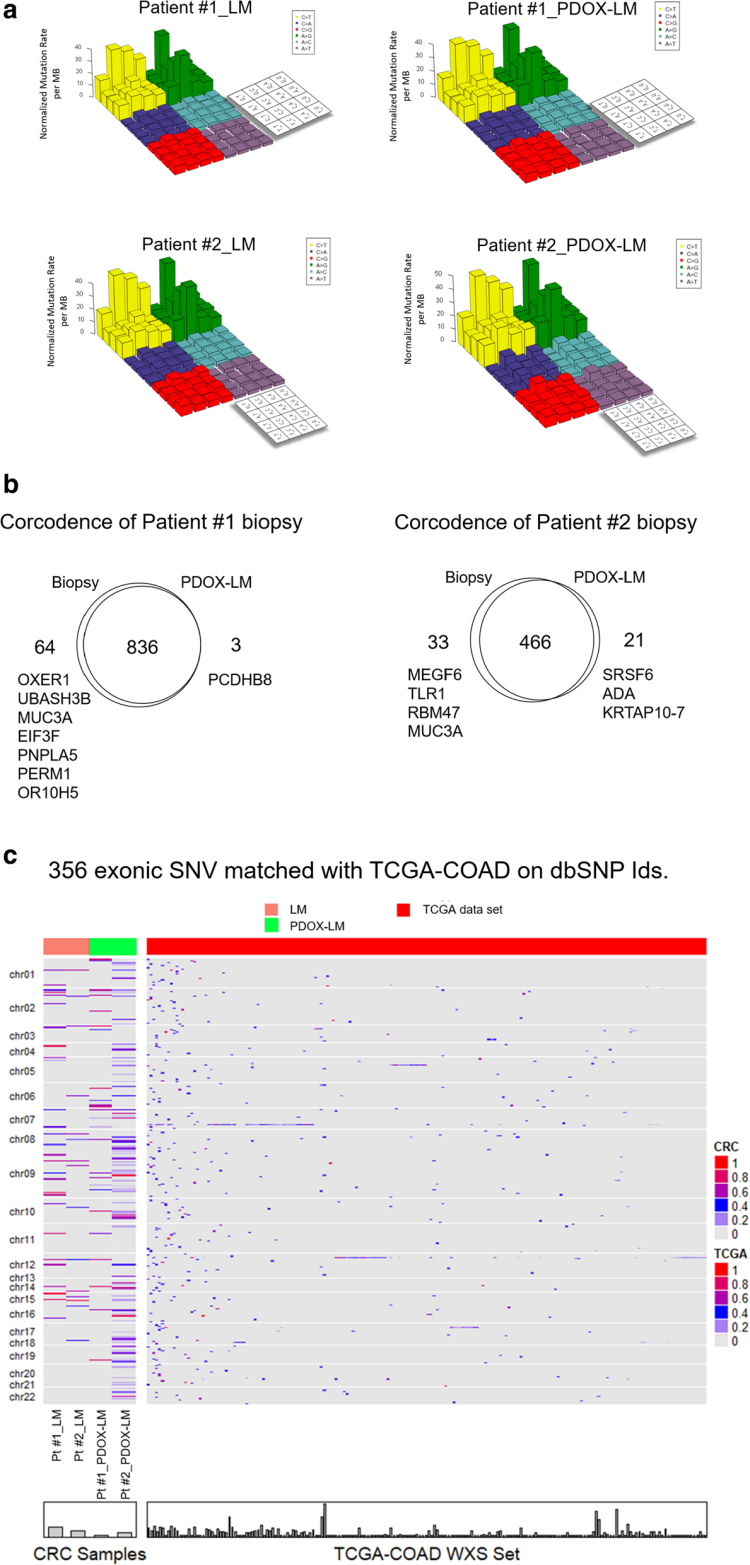
CRC Subtypes and mutation rate in PDOX-LM models and Corresponding Biopsy liver metastases Specimens. **a** Lego plots of sequencing data across donor liver metastases (LM) and xenografted liver metastases (PDOX-LM) samples indicates the potential structural changes of transitions. Lego plot of sequencing data derived from CRC samples shows preponderance of CpG transitions, C > T and A > G, accompanied by sporadic transversions, such as C > G, C > A are observed in either selected top variants or the variants identified by the sequencing. **b**, **c** Venn diagram showing the concordance of driver mutations found in colorectal liver metastases biopsy and PDOX-LM model in Patient #1 (B) and #2 (C). Somatic variants within the coding regions in xenografted liver metastases showed 0.96 and 0.90 frequency of concordance in the corresponding donor liver metastases in Patient #1 and #2, respectively. **d** Heatmap of 356 exonic SNV in donor or xenografted liver metastases sample matched with TCGA-COAD on dbSNP Ids. 158 recurrence star mutations from the publication by van de Wetering et al., 2015, Cell 161, 933–945, May 7, 2015 were matched against CRC data set, 36 matched variants were found. The clustering heatmap was generated for these 36 star mutations on 4 donor or xenografted liver metastases samples. The differential patterns are not obvious. 2361 variants from TCGA-COAD tumor data set (no normal data presented) with averaged alternate allele frequency above 0.4 were selected to generate the clustering heatmap with 4 donor or xenografted liver metastases samples. Again, the differential patterns are not obvious. The heatmap was based on 356 exonic SNV (Synonymous and Non-synonymous) in TCGA samples

Genomic DNA were isolated from liver metastases and matched normal colonic mucosa for sequencing in order to identify tumor-specific somatic mutations [25]. Genomic DNA were available for comparative analysis only for patient #1 and #2. The mutation rates per Mb varied widely for different tumor biopsy specimen (range 3.2 to 8.6), with a median value of 6.4 in the xenografted liver metastases, higher than the median rate of 3.7 in the donor liver metastases (Fig. 3a, Additional file 2: Table S1A–C). Mutations were predominantly CpG to T transitions, consistent with results from large-scale CRC sequencing [9, 16, 25]. Both xenografted liver metastases displayed non-hypermutation (≤ 10 mutations/Mb), which was consistent with the matched donor liver metastases.

Mutations known to enhance the selective growth advantage of the cancer cells, or driver mutations, were specifically examined. These driver mutations were overwhelmingly present in the xenografted liver metastases. Somatic variants within the coding regions in xenografted liver metastases were highly concordant with the corresponding donor liver metastases (Patient #1, 0.96 frequency of concordance; Patient #2, 0.90 frequency of concordance) (Fig. 3b, Additional file 2: Table S1C). Indeed, combined analysis of SCNAs and single nucleotide variants (SNVs) to infer Cancer Cell Fractions (CCF) [28, 29] in the donor and xenografted liver metastases, revealed that the common CRC driver mutations were maintained in the establishment of model (Additional file 2: Table S1D).

We showed that both xenografted and matched donor liver metastases shared many missense growth promoting mutation, such as *TP53* (exon3,G > A), *POLE* (exon8,C > A), *AXIN2* (exon 7 G > C; exon 2 G > A), *TCF7L2* (exon13 C > A) and *RNF43* (exon2 C > T) alterations, suggesting they arose from the same somatically altered progenitor cell (Additional file 2: Table S1B). In brief, the most commonly altered genes in donor biopsy [25, 30, 31] were well represented in the xenografted liver metastases (Fig. 3b). Inactivating alterations to the tumor suppressors *APC*, *TP53*, *FBXW7* and *SMAD4*, as well as activating mutations in *KRAS* (codon 12) were observed (Additional file 2: Table S1B) [25]. Interestingly, the successful establishment of both *KRAS* mutant type and *KRAS* wild type organoids implied that *KRAS* status did not influence the establishment of PDOX-LM models. On the other hand, discordant mutations were also assessed for their biological significance in cancer, based on Cancer Gene Census and data reported from the PanCancer analysis of 5000 whole exomes [31, 32]. Only 3.9% (14/356) of discordant mutations found in xenografted liver metastases affected cancer-associated genes, including a third hit to *APC*, which was already biallelically inactivated in Patient #1 samples, and *MEGF6* mutation in Patient #2 (Fig. 3b, c, Additional file 2: Table S1E). The discordant mutations had a mean allelic frequency of 7.4% and 10.4% for the donor and xenografted biopsy, respectively.

Mutations of genes in DNA mismatch repair (MMR)-associated pathways has been reported to associate with a hypermutated phenotype [33]. We showed that missense mutations were absent in *MSH3* in both cases, consistent with their clinical classification as non-hypermutated CRC cases [25]. However, the limited sample size did not allow a statistical analysis for somatic copy number alterations to identify significant regions of amplification and deletions.

In aggregate, our xenografted liver metastases faithfully captured the genomic features of the donor liver metastases.

### Transcriptome analysis of donor and xenografted liver metastases

**Fig. 4:**
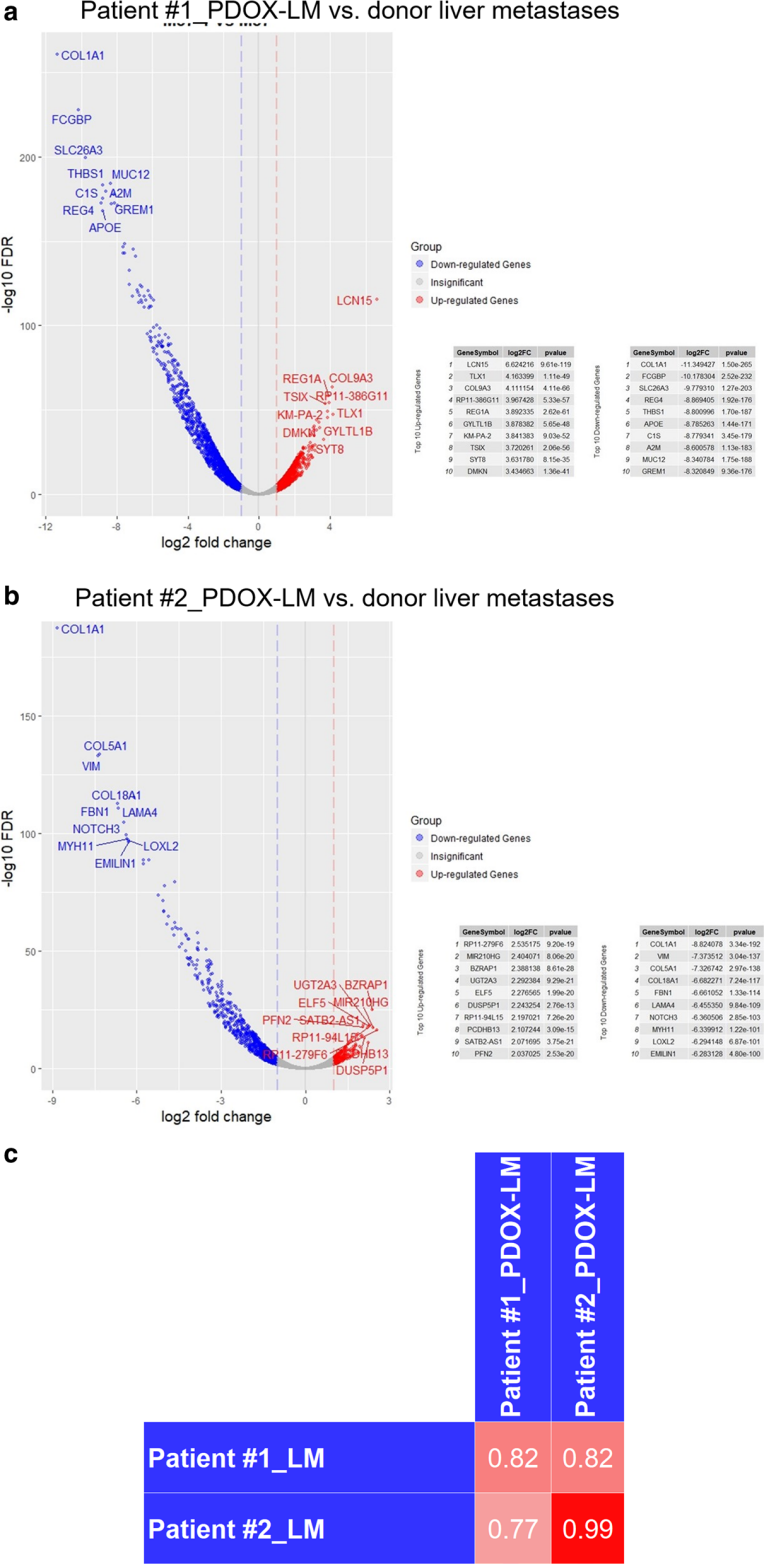
Gene expression between donor and xenografted liver metastases. **a**, **b** Differential expressed genes between donor and matched xenografted liver metastases samples of patient #1(A) and #2 (B) Top 10 up-regulated genes and down-regulated genes in patient #1 and #2 were listed in tables above. Multiple genes with similar patterns of expression (fold change < 4) were also listed in both matched samples, such as *TP53*, *KRAS*, *NRAS*, *EGFR*, *ERBB2*, *APC*, *CTNNB1*, *AXIN2*, *SMAD4* and *CDK2*. **c** Pearson’s correlation between donor liver metastases and xenografted liver metastases samples based on the RNA-seq data of driver genes. Pearson’s correlation coefficient in patient #1(r = 0.82, p-value = 0.001) and #2 (r = 0.99, p-value = 0.001) were showed. LM, liver metastases; PDOX-LM, patient-derived organoids-based xenografted liver metastases

The murine livers provide the similar mesenchyme, blood vessels, hepatic stellate cell, etc. And donor and xenografted liver metastases were formed under closely resemble condition. Therefore, we assumed that this model allows for direct gene expression analysis of subclone with liver affinity. Transcriptomic characterization was analyzed using Affymetrix single transcript arrays. Figure 4a, b showed the volcano plots of differentially expressed genes. Both donor and xenografted liver metastases exhibited high heterogeneity. Top 10 up-regulated genes and down-regulated genes in patient #1 and #2 were also listed (Fig. 4a, b). The most of up- or down-regulated genes were not common CRC driver genes except *NOTCH3* and *MYH11*. Collagen-associated genes, including *COL1A1*, *COL5A1*, *COL18A1* and *COL9A3* were examined in top 10 up- or down-regulated genes (Fig. 4a, b), which attributed to less stoma composition in xenografted liver metastases than that of matched donor. Next, we searched for genes co-expressed between donor and xenografted liver metastases. Both donor and xenografted liver metastases co-expressed driver genes including *TP53*, *KRAS*, *NRAS*, *EGFR*, *ERBB2*, *APC*, *CTNNB1*, *AXIN2*, *SMAD4* and *CDK2* (fold change < 4) (Table 3) [34]. Indeed, large sample analysis are warranted to confirm whether xenografted liver metastases faithfully capture the expression of CRC driver gene in donor liver metastases.

**Table 3:** Genomic and Transcriptomic Characterization of PDOX-LM models mimicking the donor liver metastases, Related to Fig. 5

Gene symbols	Log2FC	*P*
Patient #1 PDOX-LM vs. donor liver metastases		
*CDX2,TP53,POLE*, *AXIN2*, *CDK2*, *CDK4*, *AKT1*	0 to 2	NA
*IGF2R*, *APC*, *MDM2*, *KRAS*, *EGFR*, *CTNNB1*, *CDK6*, *SMAD4*, *BRAF*, *ARID1A*, *MTOR*, *SMAD2*, *BCL3*, *PI3KCA*, *FBXW7*, *MAPKAP1*, *ERBB2*, *ERBB3*, *AKT2*, *TCFL2*, *NRAS*	− 2 to 0	NA
Patient #2 PDOX-LM vs. donor liver metastases		
*NRAS*, *KRAS ERBB3*, *EGFR*, *AXIN2*, *CDK4*, *BRAF*, *APC*, *FBXW7*, *TCF7L2*, *TP53*, *CDK2*, *MAP2K4*, *IGF2R*	0 to 2	NA
*IGF1R*, *MTOR*, *CDK6*, *AKT1*, *ARIDA1A*, *MDM2*, *AKT2*, *SMAD4*, *PI3KCA*, *ERBB2*, *CTNNB1*	− 2 to 0	NA

### Drug response-associated mutations and gene expression

**Fig. 5:**
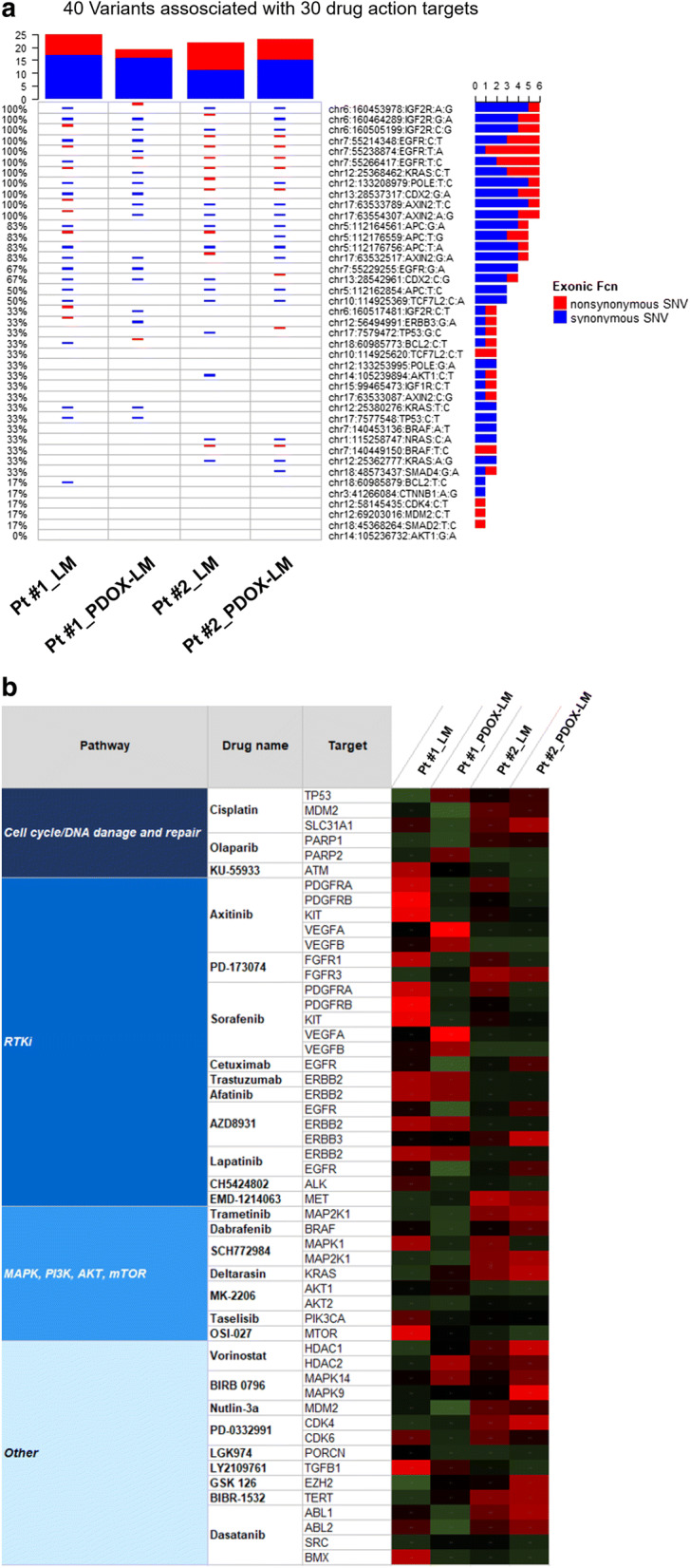
Drug response-associated mutations and gene expression. **a** 40 variants are associated with 30 drug action targets. The most variable variants (top 40 most variable) were summarized, different color tags represented different exonic variant subtypes. Multiple genes, including *IGF2R*, *EGFR*, *POLE*, *AXIN2*, *APC*, *CDX2*, *ERBB3* and *TP53*, were completely identical in both donor and xenografted liver metastases. In contrast, mutations status in *IGF1R*, *KRAS*, *NRAS*, *BRAF*, *TCF7L2*, *ERBB3*, *TP53*, *MDM2*, *BCL2*, *AKT*, *SMAD2*, *SMAD4*, *CTNNB1* and *CDK4* were partially different. **b** Heatmap of gene expression associated with 30 drug action targets A total of 30 drug response-associated target gene were summarized. Both donor and xenografted liver metastases may demonstrated identical drug response to cisplatin, olaparib, PD-173074, cetuximab, trastuzumab, afatinib, AZD8931, lapatinib, trametinib, SCH772984, EMD-1214063, deltarasin, MK-2206, nutlin-3a, PD-0332991, LGK974, LY2109761, BIRB-1532 and dasatanib. In contrast, donor and xenografted liver metastases may demonstrate different response to KU55933, axitinib, sorafenib, CH5424802, taselisib, QSI-027, vorinostat, dabrafenib, BIRB 0796 and GSK126

To identify the accordance of drug response between donor and xenografted liver metastases, we compared drug response-associated mutations and gene expression. To compare the predictive value in drug response, target genes for 30 targeted agents or chemotherapy agents were available for both xenografted and matched liver metastases. The analysis included 20 genes identified as mutant, or frameshift or non-frameshift insertion as described by previous reports (Fig. 5a) [35, 36]. *KRAS* and *NRAS* status were detected the same between donor and xenografted liver metastases by WES, which is associated with the response of the anti-*EGFR* inhibitors cetuximab and BIBW2992 (afatinib) (Figs. 5a). The donor and xenografted liver metastases with mutant-type *KRAS* demonstrated a higher expression than the counterpart with wild-type *KRAS* (Figs. 5a, b, Additional file 2: Table S1B).

We also identified a number of discordant mutant-type target genes during the establishment of xenograft model, including *IGF2R*, *EGFR*, *POLE*, *AXIN2*, *APC*, *CDX2*, *ERBB3*, *TP53.* Loss-of-function mutations of the tumor suppressor *TP53* were associated with resistance to cisplatin and nutlin-3a, an inhibitor of *MDM2* (Fig. 5a, Additional file 2: Table S1B). Only xenografted liver metastases were nonsynonymous mutant-type for *TP53* by WES, which predicted a different response to cisplatin or nutlin-3a between donor and xenografted liver metastases. Similarly, donor and xenografted liver metastases demonstrated distinct sensitivity to the pan-*ERBB* inhibitor AZD8931 and the chemotherapeutic gemcitabine.

We also performed a validation screen in transcriptome analysis and compared expression of responses-associated genes between donor and xenografted specimens (Fig. 5b). A total of 30 drug response-associated target genes were summarized in Fig. 5b. Both donor and xenografted liver metastases demonstrated identical drug response to cisplatin, olaparib, PD-173074, cetuximab, trastuzumab, afatinib, AZD8931, lapatinib, trametinib, SCH772984, EMD-1214063, deltarasin, MK-2206, nutlin-3a,PD-0332991, LGK974, LY2109761, BIRB-1532 and dasatanib. In contrast, donor and xenografted liver metastases demonstrated different response to KU55933, axitinib, Sorafenib, CH5424802, taselisib, QSI-027, vorinostat, dabrafenib, BIRB 0796 and GSK126 (Fig. 5b).

### PDOX-LM models mimicking clinical drug response of chemotherapy

**Fig. 6:**
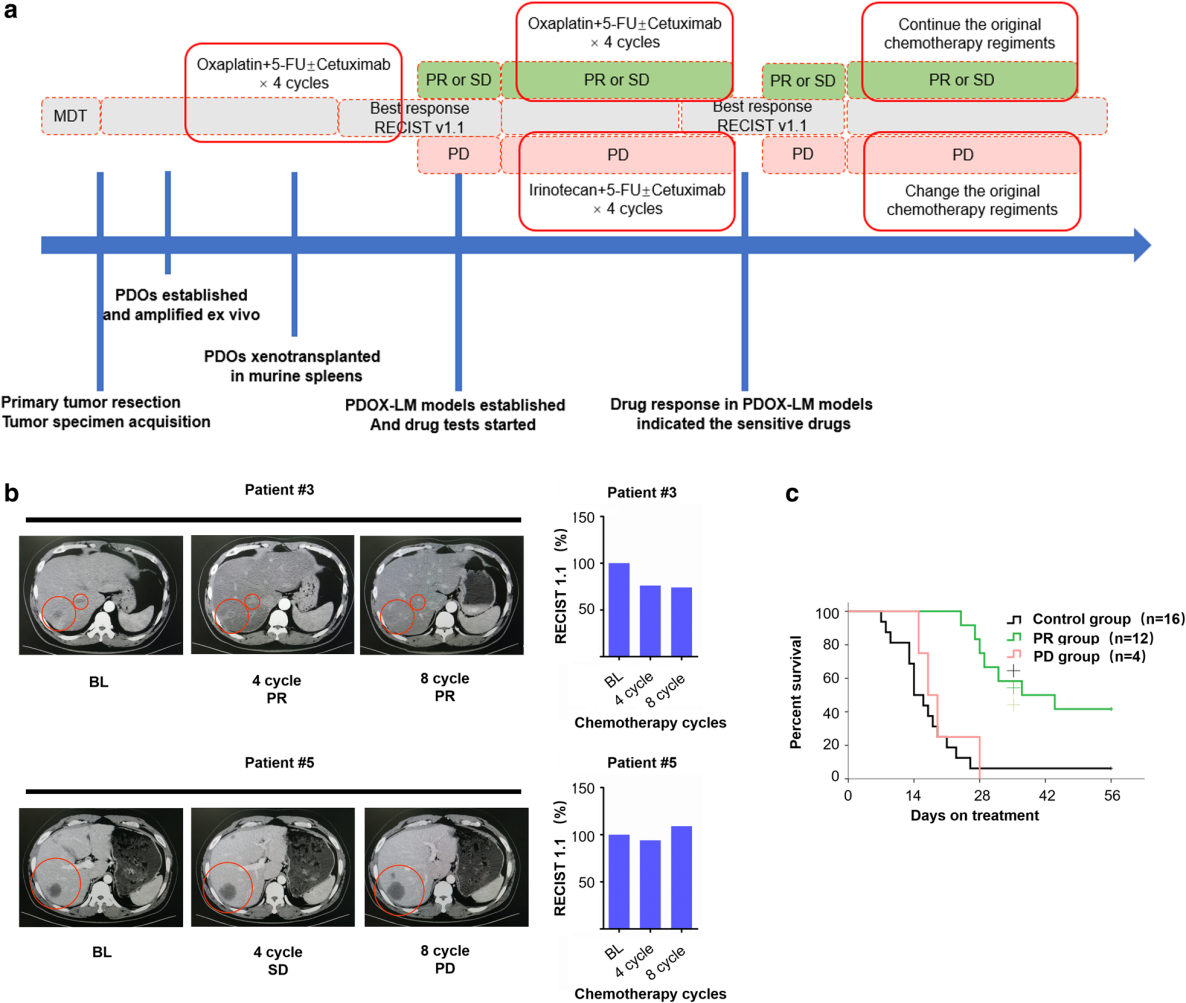
PDOX-LM model mimic patient’s response to chemotherapy. **a** Summary of clinical history of donor patient with initially unresectable liver or lung metastases. The clinical course of the patients with unresectable metastases accepting conversion therapy is summarized. Red-lined boxes indicate periods of administration of the indicated therapeutic regiments. Blue vertical lines indicate timing of tumor specimen acquisition from surgical procedures, PDOs model or PDOX-LM model establishment as well as dates of drug test starting and drug-response assessed by overall survival of murine model. PR, partial response, PD, progressive disease; according to RECIST (Response Evaluation Criteria in Solid Tumors) 1.1. **b** PDOX-LM models were generated from primary tumor biopsies of patients with unresectable liver metastases (patient #3 and #5, red circles in the bottom panel) that showed initial response to mFOLFOX6 regiments. Violet bars indicate overall tumor volume [according to RECIST 1.1], and red bars indicate volume of the target metastasis. **c** Kaplan–Meier survival curve showing a significant increase in the survival of PR group compared to both PD group and control group. NOTE: PR group: PDOX-LM models derived from the donor patients with a clinical drug response of PR (RECIST 1.1) were tested for oxaliplatin and 5-Fu. PD group: PDOX-LM models derived from the donor patients with a clinical drug response of PD (RECIST 1.1) were tested for the combination of oxaliplatin and 5-Fu. NS: Not significant, p-value ≥ 0.05, ***p-value ≤ 0.001

In order to verify if our PDOX-LM models reliably recapitulate patients’ response to clinical drugs, we looked for established models in which the corresponding donor patients had been treated with mFOLFOX6 chemotherapy (Oxaliplatin and 5-FU were both key component). And this PDOX-LM models can predict the drug response of clinical chemotherapy treatment (Fig. 6a). Patient #3, #4, #5 and #6 were used in drug response testing, showing primary resistance to chemotherapy (Patient #3, #4, #6: partial response; Patient #5: progressive disease; according to RECIST 1.1 criteria; Fig. 6b). PDOX-LM models were divided into the control group (n = 16), PR group (n = 12) and PD group (n = 4). The overall survival (OS) of PR group were significantly prolonged (Fig. 6c), which was closely recapitulated the clinical drug response of the donor patients.

## Discussion

In the present study, we established 6 well-characterized PDOX-LM models. We combined organoid culture and in vivo splenic injection technology to obtain a rapid formation of metastatic xenografts in murine liver. As previous reports, in vivo models mimicking metastases have proven more informative than subcutaneous grown models in CRC [5, 37, 38], with improved microenviroment-metastases interaction [12–14], and evaluation of drug efficacy [39, 40]. And this study firstly revealed a rapid way to establish in vivo metastatic models based on primary tumor derived organoids with a high success rate. This PDOX-LM models could faithfully recapitulate molecular profiles and metastatic behavior of the metastatic tumors in mCRC patients.

We used organoid culture to closely recapitulate the genetic and morphological heterogeneous composition of the original tumor, offering great promise as a pre-clinical cancer model. According to the reports by Van de Wetering et al. [16], PDOs gave rise to large numbers of different organoids (10–1000), suggesting that the heterogeneous composition of the original tumor was largely conserved. In line with the previous results, we demonstrated that the organoids derived from mCRC presented a more vigorous reproductive capacity than those from non-metastatic CRC. Specifically, our results indicated a high density of organoids (100,000 cells/ml) may be an important contributing factor for the establishment of a PDOX-LM model. This may hasten more cancer stem cells and account for a successful establishment of in vivo metastatic model based on primary tumor-derived organoids.

The PDX approach has shown its high degree of translatability to the derived patient and offer in vivo models to study drug resistance and design novel treatment schedules in pre-clinical researches previously. However, it is reported that CRC PDX model require 6–8 months of propagation to be useful for treatment, making PDX model unsuitable for timely prediction for sensitive drugs [8]. And no more than 5% of the patient-derived liver metastases were successfully formed in murine model [5, 41], because most mice died of giant xenografts in situ before the metastases occurred. And the successful xenografted liver metastases took quite a longer period (about 6 months since transplantation). Even in excellent work by Arianna Fumagalli [42], their murine models also have some limitations. The liver metastases were high frequently accompanied by lung metastases in murine model. All PDOs need further editing in cancer driver gene to enhance invasiveness before transplantation, which caused high cost and are not easy to master the technology. Our PDOX-LM models have overcome those limitations, because it possessed the characteristics such as liver specific metastases, without gene editing, and simple operation for establishment. The establishment of PDOX-LM models took a mean period of 7 weeks, equivalent to the length of time to establishing CRC cell lines-based live metastases model. In addition, unlike subcutaneous xenografts, an increase of CEA levels was detectable in murine serum during the development of xenografted liver metastases [43]. Therefore, our model can mimic the biological behavior of human CRC in murine blood, facilitating the dynamical monitor of metastatic xenografts.

The present study further confirmed that the PDOX-LM could recapitulate molecular and phenotypic heterogeneity of donor liver metastases. It has been well established that Wnt and MAPK pathways account for CRC progression and metastases [13, 25, 27], and the presence o*f APC*, *KRAS*, *BRAF*, *PIK3CA*, *SMAD4* and *TP53* mutations closely relates to treatment decision and prognosis. The sequencing data verified that a similar genomic alteration in *KRAS*, *BRAF*, *PIK3CA* and *SMAD4* in both xenografted and donor liver metastases. It suggested that the common maintenance of subclone comprising major driver gene mutations during tumor progression in donor tumor or mice model [5]. Interestingly, xenografted and donor liver metastases have discordant driving alterations in *APC* and *TP53*. It is very likely that the newly established model exhibit different degrees of fitness and pathway activity because of the aggressive organoids subpopulations had greater survival rates in vivo.

Furthermore, the transcriptome analysis verified that the PDOX-LM model could replicate expression of driver genes of the donor liver metastases. High similarity RNA profiles were observed between human donor and xenografted liver metastases. Especially for driver genes, such as *APC*, *KRAS*, *BRAF*, *PIK3CA*, *SMAD4* and *TP53*, donor and xenografted liver metastases demonstrated highly consistent in both cases. According to Fang et al. [44], metastatic lesions are enriched in mutations of genes affecting *PI3K*-*Akt* signaling, cell adhesion and stellate-cell activation in the liver, the predominant metastasis site in patients. In this study, donor and xenografted liver metastases were highly consistent in expression of these genes, which means metastatic xenografts may narrow the difference in some cancer driver mutations in donor liver metastases.

In view of clinical application, we hypothesized that the PDOX-LM model can help screen the sensitive drugs. Given the complex molecular makeup of the tumor, it is important to identify the sensitive regiments to achieve a better treatment outcome.

Thus, using the WES and transcriptome analysis, we explored the power of PDOX-LM model to predict the drug response. According to ESMO guideline, cetuximab is used as the first-line target agent for mCRC, and our models could mimic the drug response of donor liver metastases by examining *KRAS* mutation status and expression level. Furthermore, it could exhibit similar response in multiple drugs, such as trastuzumab, afatinib, AZD8931, lapatinib, trametinib and SCH772984, which was due to the similar mutation status and expression in these drug-associated gene. However, our models failed to mimic the response of axitinib, sorafenib, taselisib and dabrafenib. One reason for this failure is that donor and xenografted liver metastases may partly root from different subclones of the identical primary tumor [16]. And another reason is that only tumor epithelial, but not mesenchymal niche, were maintained during the culture of organoids.

To verify whether our model can mimic the drug response in clinical tumor, we also performed the drug response testing. It showed that PDOX-LM models indeed recapitulated the clinical drug response to mFOLFOX6 in the donor patients. Notably, according to Ooft SN et al. [9], the PDO culture unable to predict the response of the combination oxaliplatin-5FU. And the absence of stromal cell in the PDO culture may partially account for this result [6, 9]. The existence of stromal cells compromise the anti-cancer effect of the cytotoxic chemotherapy [6, 45–49]. In our PDOX-LM models, the PDOX involved stromal cells in tumor xenografts development in vivo, so our PDOX-LM models could recapitulate the response of oxaliplatin and 5-Fu in donor tumor. Our drug testing results were similar as the published PDX model based on patient-derived colon cancer cells by Isabel Puig et al. [50]. Moreover, the rapid establishment of PDOX-LM models could increase the possibility of translational clinical application.

Indeed, this study has some limitations. First, more tumor samples should be included to offer further insight into the homogeneity and heterogeneity between donor and xenografted liver metastases. Second, targeted agents need to be explored by this model. Moreover, more biomarker, such as circulating tumor cells or DNA, could be verified in further by testing the murine blood.

## Conclusions

In conclusion, the PDOX-LM models, reflecting the pheno- and genotype of the original patient samples, facilitate the rational selection of appropriate metastatic models for future drug experiments. This experimental approach could reduce the high failure rate observed in early preclinical trials in mCRC, which have no regard for the biological or molecular grounds when selecting experimental drugs or the patient cohort. And it is possible that advanced genetic analysis will discover novel genetic alterations accounting for the malignant progression and drug response.

## Supplementary information

**Additional file 1: Figure S1.** Schematic depicting organoid xenotransplantation by splenic injection and comparing the homogeneity and heterogeneity between the PDOX-LM models and donor liver metastases.

**Additional file 2: Table S1.** A. 356 exonic SNV matched with TCGA-COAD on dbSNP Ids in PDOX-LM models. B. 36 Recurrent mutations in PDOX-LM models. C Somatic mutation rates in PDOX-LM models. D Cancer Cell Fractions rates; E. Discordant mutation between xenografted and donor liver metastases. A–E related to Figs. 3 and 5.

**Additional file 3: Additional methods and Table S2.** Additional methods. **Table S2.** Primary antibodies used for IF or IHC staining.

## Data Availability

The authors declare that all the data supporting the findings of this study are available within the article.

## Abbreviations

CRC: Colorectal cancer
mCRC: Metastatic colorectal cancer
CLMs: Colorectal liver metastases
PDO: Patient-derived organoids
PDOX-LM model: Patient-derived organoids-based xenografted liver metastases model
Pt: Patient
CEA: Carcinoembryonic antigen
H&E staining: Hematoxylin and eosin staining
IF staining: Immunofluorescence staining
IHC staining: Immunohistochemical staining
CCF: Cancer Cell Fractions
MMR: Mismatch repair
RECIST: Response Evaluation Criteria in Solid Tumors
PR: Partial response
SD: Stable disease
PD: Progressive disease
OS: Overall survival
