# Supplementary figures and images for: A novel patient-derived organoids-based xenografts model for preclinical drug response testing in patients with colorectal liver metastases
Source: J Transl Med. 2020 Jun 12;18:234. doi: 10.1186/s12967-020-02407-8 (PMC7291745; doi:10.1186/s12967-020-02407-8)

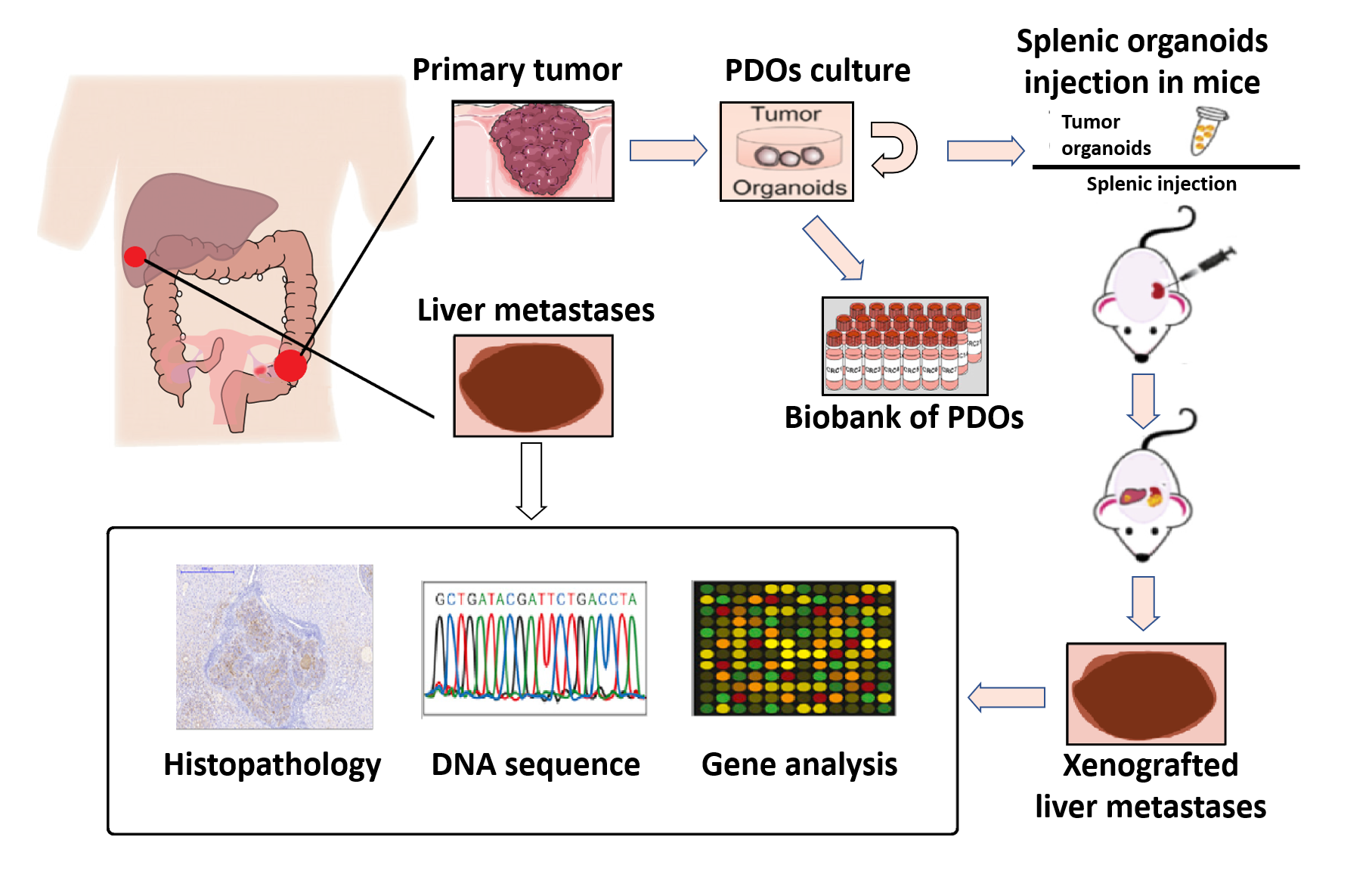

Supplement: Supplementary file 1 — Additional file 1: Figure S1. Schematic depicting organoid xenotransplantation by splenic injection and comparing the homogeneity and heterogeneity between the PDOX-LM models and donor liver metastases. [file 12967_2020_2407_MOESM1_ESM.tif]
