# Supplementary material for: A novel patient-derived organoids-based xenografts model for preclinical drug response testing in patients with colorectal liver metastases
Source: J Transl Med. 2020 Jun 12;18:234. doi: 10.1186/s12967-020-02407-8 (PMC7291745; doi:10.1186/s12967-020-02407-8)
Supplement: Supplementary file 3 — Additional file 3: Additional methods and Table S2. Additional methods. Table S2. Primary antibodies used for IF or IHC staining. [file 12967_2020_2407_MOESM3_ESM.docx]

**Additional Methods**

**Tumor isolation**

Tumors were cut into pieces and three parts were processed for IHC staining, DNA isolation and RNA isolation. The remainder was cut into smaller pieces and incubated with Collagenase II (1,5 mg/ml, Gibco), Hyaluronidase (20 ug/ml, Sigma) and Ly27632 (10 μM, Sigma) for 30 min at 37 ℃ while shaking. After incubation, fetal calf serum (FCS, Gibco) was added and the mixture was put over a 100-μm cell strainer to remove large fragments. Cells were subsequently spun at 1,000 rpm for 3 min. The pellet was resuspended in basal culture medium and spun again at 1,000 rpm. The procedure was repeated twice to remove debris and collagenase. The tumor material was resuspended in Matrigel (BD bioscience, No.356331) and plated at different densities.

**Organoids culture**

Tumor tissue-derived organoids were cultured in Human Intestinal Stem Cell (HISC) medium. The composition of HISC medium is: advanced Dulebecco’s modified Eagle medium/F12 supplemented withpenicillin / streptomycin (Gibco), 10mM HEPES and Glutamax (Gibco), 20% R-Spondin 1 conditioned medium (Peprotech), 10% Noggin conditioned medium (Peprotech), 1× B27 (Gibco), 1,25 mM n-Acetyl Cysteine (Sigma), 10 mM Nicotinamide (Sigma), 50 ng/ml human EGF (Peprotech), 10 nM Gastrin (Tocris), 500 nM A83-01(Tocris), 3 μM SB202190 (Sigma), 10 nM Prostaglandin E2 (Tocris), and 100 mg/ml Primocin (Invivogen) [1].

**Passage of organoids**

Organoids culture medium was refreshed every two days. To passage the organoids, Matrigel was broken up by pipetting and organoids were collected in a tube. The organoids were centrifuged at 1,000 rpm for 3 min and the medium removed. 5 ml Triple Express (Invitrogen) was added and the organoids were incubated at 37 ℃ for approximately 5 min. Every minute, a visual check was done to verify the size or the organoids. Care was taken not to treat the organoids to long with Triple Express. FCS and medium were added and cells were spun down at 1,500 rpm for 3 min. The pellet was taken up in Matrigel and cells were plated in droplets of 5-10 ml each. After allowing the Matrigel to solidify, HICS medium (for PDOs), supplemented with 10 M LY27632 (Sigma), was added to the plates and organoids were incubated at 37℃.

**Histology procedures**

Organoids were observed using live confocal image by IF staining. Tissues and organoids were fixed in 10% neutral buffered formalin and embedded in paraffin. Sections were subjected to H&E as well as IHC staining. The following primary antibodies were used for IF staining: anti-Lgr5 antibody, anti-E-cadherin, Hoechst 33342 (Cas.no. 23491-52-3), 10μg/ml. The following primary antibodies were used for IHC staining: anti-CEA antibody, anti-HER2 antibody, anti-Ki67 antibody, anti-Cytokeratin 20 antibody, anti-EGFR antibody, anti-VEGF antibody, anti-FGFR2 antibody, anti-VEGFR2 antibody (Table S2).

All staining experiments were repeated at least 3 times. Positive cells were scored irrespective of the staining intensity. The percentage of positive cells was semi-quantitatively scored by two independent persons.

Table S2 Primary antibodies used for IF or IHC staining.

| Item | Corporation & item no. | Diluted concentration |
| --- | --- | --- |
| anti-Lgr5 antibody | Abcam Ab71225 | 1:100 |
| anti-E-cadherin antibody | Abcam Ab1416,1:100 | 1:100 |
| anti-CEA antibody | Proteintech 10421-1-AP | 1:100 |
| anti-HER2 antibody | Proteintech 18299-1-AP | 1:100 |
| anti-Ki67 antibody | Proteintech 27309-1-AP | 1:200 |
| anti-Cytokeratin 20 antibody | Proteintech 17329-1-AP | 1:100 |
| anti-EGFR antibody | Proteintech 18986-1-AP | 1:100 |
| anti-VEGF antibody | Proteintech 19003-1-AP | 1:50 |
| anti-FGFR2 antibody | Proteintech 13042-1-AP | 1:50 |
| anti-VEGFR2 antibody | Proteintech 26415-1-AP | 1:50 |

**Whole-exome sequencing and single nucleotide polymorphisms**

For each sample, 250 ng of DNA was sheared and subject to whole-exome sequencing using the Agilent v2 capture probe set and sequenced by HiSeq2500 using 76 base pair reads, as previously described [2,3]. A median 12.4 Gb of unique sequence was generated for each sample. Sequence data were locally realigned to improve sensitivity and reduce alignment artifacts prior to identification of mutations, insertions, and deletions as previously described [4-6].

**MANOVA genomic input**

Only genes that have been identified as being mutated in > 10% of colorectal cancers were included [7]. We ensured that non-synonymous variants identified for these genes were variants present in the clinical population by comparing the organoid variants with the mutations present in the COSMIC database (http://cancer.sanger.ac.uk/cosmic/).

**Variant calling**

Aligned sequence data were locally realigned to improve sensitivity and reduce alignment artifacts prior to identification of mutations, insertions, and deletions were called using MuTect and Indelocator as previously described [5,4]. When no normal organoids culture was derived, the mutation calls for the tumor samples were filtered against a panel of normal made up of 1,000 exomes. To establish concordance of mutations and remove false-positives due to coverage, alterations that were covered by greater than 10 reads or had an allelic fraction of greater than 5% in biopsy and tumor organoid were used in Figure 3. All discordant reads were than manually reviewed in Integrated Genome Viewer to rule out sequencing or alignment artifacts.

**Somatic copy-number analysis**

Somatic copy-number analysis was performed using segmented copy-number profiles generated from whole-exome sequencing using the SegSeq algorithm [8]. Briefly, read depth in tumor and normal pairs was calculated to provide relative copy-number ratios at each exon followed by circular binary segmentation [9]. The GISTIC2.0 algorithm [10] was used to investigate focal and arm-level copy-number changes (Table S1A-S1E).

**Cancer cell fraction**

Cancer cell fraction analysis was performed as previously described [11,12]. Briefly, the ABSOLUTE algorithm was used to establish purity, ploidy and DNA copy number for each sample [11]. Each SNV was considered clonal or subclonal if the calculated probability exceeded 0.95.

**RNA sequence data processing**

RNA was extracted using QIAGEN RNA mini kit according to the manufacturer procedures (QIAGEN). RNA from 2 CRC donor and matched xenografted liver metastases samples was hybridized on Affymetrix Human Gene 2.0 ST arrays. The raw CEL files were processed with Affymetrix Power Tools using the Hg19 genome build and NetAffx annotation dating from 09-30-2018. Between-array normalization was performed using rma-sketch, within APT. This resulted in an intensity matrix of 21,681 genes by 2 samples. For analysis of individual genes, data were analyzed using the R2 web application, which is freely available at http://r2.amc.nl.

**Transcriptome Analysis**

For mRNA sequencing, tumor and xenografts samples were hand-picked and deposited into trizol (life technologies). The RNA was extracted using chloroform and precipitated with iso-propanol. The RNA-pellet was then processed using the CEL-seq protocol[13] and sequenced on an Illumina Nextseq using 75bp paired end sequencing. After sequencing, read 1 was aligned to the hg19 RefSeq human transcriptome downloaded from the UCSC genome browser [14] using bwa (Li and Durbin, 2010) with default parameters [15]. Read 2 contains a barcode identifying the sample from which the read originated CEL-seq only sequences the most 30 prime end of a transcript and generates one read per transcript. Samples with less than 500.000 reads were discarded after which the remaining samples were rpm normalized.

**TCGA data**

From TCGA we downloaded Level 3 RNA-seq data, frozen tissue, labeled COAD (Colon adenocarcinoma), from both the IlluminaGA and IlluminaHiSeq platforms. This corresponds to 41 normal samples and 431 tumor samples. While these data are ready for analysis, we noticed a strong batch effect between the two platforms (IlluminaGA and IllumnaHiSeq) and adjusted for that when combining all samples. This is described in more detail in the next section.

**Combining data sets**

Before combining, both data sets, donor and xenografts tumor tissues and TCGA RNA-seq, were log-transformed and mean-centered. The RNA-seq IlluminaGA and IlluminaHiSeq are mean-centered independently. Next, we took advantage of the fact that there are normal samples in both data sets. We used ComBat [16] to normalize all samples together by specifying the data type as the batch and adding the normal/tumor assignment as an additional factor. Then we extracted only the normalized tumor samples for subtyping.

References:

1. van de Wetering M, Francies HE, Francis JM, Bounova G, Iorio F, Pronk A et al. Prospective derivation of a living organoid biobank of colorectal cancer patients. CELL. 2015;161:933-45.

2. Fisher S, Barry A, Abreu J, Minie B, Nolan J, Delorey TM et al. A scalable, fully automated process for construction of sequence-ready human exome targeted capture libraries. GENOME BIOL. 2011;12:R1.

3. Imielinski M, Berger AH, Hammerman PS, Hernandez B, Pugh TJ, Hodis E et al. Mapping the hallmarks of lung adenocarcinoma with massively parallel sequencing. CELL. 2012;150:1107-20.

4. Ojesina AI, Lichtenstein L, Freeman SS, Pedamallu CS, Imaz-Rosshandler I, Pugh TJ et al. Landscape of genomic alterations in cervical carcinomas. NATURE. 2014;506:371-5.

5. Cibulskis K, Lawrence MS, Carter SL, Sivachenko A, Jaffe D, Sougnez C et al. Sensitive detection of somatic point mutations in impure and heterogeneous cancer samples. NAT BIOTECHNOL. 2013;31:213-9.

6. DePristo MA, Banks E, Poplin R, Garimella KV, Maguire JR, Hartl C et al. A framework for variation discovery and genotyping using next-generation DNA sequencing data. NAT GENET. 2011;43:491-8.

7. Lawrence MS, Stojanov P, Mermel CH, Robinson JT, Garraway LA, Golub TR et al. Discovery and saturation analysis of cancer genes across 21 tumour types. NATURE. 2014;505:495-501.

8. Chiang DY, Getz G, Jaffe DB, O'Kelly MJ, Zhao X, Carter SL et al. High-resolution mapping of copy-number alterations with massively parallel sequencing. NAT METHODS. 2009;6:99-103.

9. Olshen AB, Venkatraman ES, Lucito R, Wigler M. Circular binary segmentation for the analysis of array-based DNA copy number data. BIOSTATISTICS. 2004;5:557-72.

10. Mermel CH, Schumacher SE, Hill B, Meyerson ML, Beroukhim R, Getz G. GISTIC2.0 facilitates sensitive and confident localization of the targets of focal somatic copy-number alteration in human cancers. GENOME BIOL. 2011;12:R41.

11. Carter SL, Cibulskis K, Helman E, McKenna A, Shen H, Zack T et al. Absolute quantification of somatic DNA alterations in human cancer. NAT BIOTECHNOL. 2012;30:413-21.

12. Landau DA, Carter SL, Stojanov P, McKenna A, Stevenson K, Lawrence MS et al. Evolution and impact of subclonal mutations in chronic lymphocytic leukemia. CELL. 2013;152:714-26.

13. Yanai I, Hashimshony T. CEL-Seq2-Single-Cell RNA Sequencing by Multiplexed Linear Amplification. Methods Mol Biol. 2019;1979:45-56.

14. Meyer LR, Zweig AS, Hinrichs AS, Karolchik D, Kuhn RM, Wong M et al. The UCSC Genome Browser database: extensions and updates 2013. NUCLEIC ACIDS RES. 2013;41:D64-9.

15. Li H, Durbin R. Fast and accurate long-read alignment with Burrows-Wheeler transform. BIOINFORMATICS. 2010;26:589-95.

16. Johnson WE, Li C, Rabinovic A. Adjusting batch effects in microarray expression data using empirical Bayes methods. BIOSTATISTICS. 2007;8:118-27.
